# Spray Drying of *Rhodomyrtus tomentosa* (Ait.) Hassk. Flavonoids Extract: Optimization and Physicochemical, Morphological, and Antioxidant Properties

**DOI:** 10.1155/2014/420908

**Published:** 2014-12-04

**Authors:** Pingping Wu, Qian Deng, Guangzhi Ma, Nianghui Li, Yanyan Yin, Baojun Zhu, Meiling Chen, Ruqiang Huang

**Affiliations:** ^1^College of Life Science, South China Normal University, Guangzhou 510631, China; ^2^Milne Fruit Products, Inc., 804 Bennett Avenue, Prosser, WA 99350, USA

## Abstract

The optimal condition of spray drying purified flavonoids extract from *R. tomentosa* berries was studied by response surface methodology. The optimized condition for microencapsulation was of maltodextrin to gum Arabic ratio 1 : 1.3, total solid content 27.4%, glycerol monostearate content 0.25%, and core to coating material ratio 3 : 7, resulting in EE 91.75%. Prepared at the optimized condition, the flavonoids extract microcapsules (FEMs) were irregularly spherical particles with low moisture content (3.27%), high solubility (92.35%), and high bulk density (0.346 g/cm^3^). DPPH radical scavenging activity of FEMs was not decreased after spray drying (*P* > 0.05) and higher than those in citric acid and rutin at the same concentration. Moreover, FEMs effectively retarded the oxidation of fresh lard during the 10-day storage period compared with vitamin C, nonencapsulated flavonoids extract, and rutin. Therefore, FEMs produced at the optimized condition could be used as powder ingredients with antioxidant capacities.

## 1. Introduction

*Rhodomyrtus tomentosa* (Ait.) Hassk. (*R. tomentosa*), also named as Rose Myrtle, is an evergreen shrub in the family Myrtaceae and mainly grown in southeast Asian countries, especially southern China, Japan, and Thailand [1]. The edible berries of* R. tomentosa* are dark violet and bell-shaped and have been historically used as a folk medicine to treat diarrhea, dysentery, and traumatic hemorrhage [2]. Our previous study has reported the total content of flavonoids of* R. tomentosa* berries and identified six of flavonoids (myricetin, quercetin, dihydromyricetin, kaempferol, quercetin 7, 4′-diglucoside, and vitexin) by UPLC-MS/MS [3]. In addition, the antioxidant capacities both* in vitro* and* in vivo* were confirmed. However, flavonoids are sensitive to environmental factors such as light, heat, pH, and O_2_ and of low water solubility [4]. After oral administration, the flavonoids undergo degradation in the drastic acidic stomach environment, resulting in reduction of their health and therapeutical benefits [5]. Therefore, improving the stability and solubility of flavonoids would hugely enhance their potential application.

Microencapsulation is a technology that envelops sensitive ingredients in a protective coating material in order to prevent these ingredients from adverse reaction, volatile loss, or nutritional deterioration [6]. Spray drying is the most prevalent technology for microencapsulation due to its higher equipment availability and cheaper operational cost [7]. The choice of coating materials in spray drying microencapsulation critically influences every stage of production (emulsification before drying and retention of bioactive and volatile compounds during drying) and product stability [8]. Gum Arabic of excellent emulsification property and maltodextrin of low viscosity and good solubility are used frequently for spray drying microencapsulation process [9, 10]. Different ratios of maltodextrin to gum Arabic and different dextrose equivalents have been widely used to encapsulate various compounds such as unsaturated fatty acids, essential oils, plant extracts, and fruit and vegetable juices [11–15]. Glycerol monostearate is one of the most important emulsifiers which provide interfacial interactions thus enhancing emulsification [16].

Therefore, in order to maintain the stability and bioactivity of flavonoids from* R. tomentosa* berries, the optimal microencapsulation condition and the antioxidant capacities of flavonoid microcapsules produced at the optimal condition were studied in this paper. This study is the first successful development on* R. tomentosa* berries flavonoids extract microcapsules.

## 2. Materials and Methods

### 2.1. Materials and Reagents

*R. tomentosa* berries were purchased from Guangzhou Medicine Market (Guangzhou, Guangdong, China). Rutin was purchased from the National Institute for the Control of Pharmaceutical and Biological Products (Beijing, China). AB-8 macroporous resin was purchased from the Chemical Plant of NanKai University (Tianjin, China). 2, 2-Diphenyl-1-picrylhydrazyl (DPPH) was purchased from Sigma Chemical Co. (St. Louis, MO, USA). Maltodextrin (MD, 5–10 DE) was purchased from Omega Biotech Ltd. (Shanghai, China). All other chemicals used for analysis were of analytical reagent grade, obtained from Guangzhou Chemical Reagent Corporation (Guangzhou, Guangdong, China).

### 2.2. Preparation of Flavonoids Extract (FE)

The air-dried fruits were powdered (40 mesh) and extracted for 4 hr twice with 95% ethanol under reflux (70°C). The combined extract was evaporated to near dryness under vacuum at 50°C. The extract redissolved in water was then extracted with petroleum ether for 2 times and the water-soluble fraction was purified by AB-8 macroporous resin eluted with 40% ethanol. The collected solution was concentrated and dried to powder format. Then the pure FE was obtained and stored at 5°C for the further usage.

### 2.3. Flavonoids Extract Microcapsules (FEMs) Preparation

The emulsion solutions were prepared according to the conditions designed in Box-Behnken design and response surface method (RSM) (Tables 1 and 2). The gum Arabic and maltodextrin were previously dissolved in distilled water (50–60°C) separately for 1 hr and then mixed together for 5 min. GSM and FE (from 2.2) were then slowly added to the coating solutions with constant stirring. The emulsified solution was further homogenized at 40 MPa for 5 min by GYB60-6S high pressure homogenizer (Donghua High Pressure Homogenizer Factory, Shanghai, China), followed by feeding into a Mini Spray Dryer SY-6000 (Shiyuan Bio. Co., Shanghai, China) with 0.7 mm diameter nozzle. According to the preliminary study, the inlet temperature was set at 150 ± 2°C and the outlet temperature was 100 ± 5°C. The pressure of compressed air for the flow of the spray was 4.0 MPa and the feeding rate was 30%. The end product FEMs were kept in self-sealing bags which were stored in a desiccated container at 5°C before analysis.

### 2.4. Analysis of Total Flavonoid Content of Microcapsules (*TFC*_*whole*_)

The modified NaNO_2_-Al(NO_3_)_3_-NaOH colorimetric method [22] was used to determine TFC_whole_. 100 mg of FEMs was dispersed in 1 mL 50% ethanol aqueous solution and subsequently mixed using a sonicator (KQ-200KDB, Shumei, China) for 15 min [23]. The mixture was filtered through a 0.45 *μ*m Millipore filter for analysis. Rutin (0–50 mg/mL) was used as a standard. TFC_whole_ was expressed as milligrams of rutin equivalents per gram of microcapsule weight.

### 2.5. Analysis of Surface Total Flavonoids Content (*TFC*_*surface*_) and Encapsulation Efficiency

To determine the TFC_surface_, 100 mg of FEMs was dispersed in 1 mL of a mix of ethanol and methanol (1 : 1, v/v) by vortexing at room temperature for 1 min, followed by filtration [23]. The TFC_surface_ was measured with the same method described in TFC_whole_ section.

Encapsulation efficiency (EE) was calculated from the following equation:

(1) $$\begin{array}{c} \text{EE}\left(\%\right)=\frac{\left(\text{TF}{\text{C}}_{\text{whole}}-\text{TF}{\text{C}}_{\text{surface}}\right)}{\text{TF}{\text{C}}_{\text{whole}}}\times 100, \end{array}$$

where TFC_whole_ was the total amount of flavonoids in FEMs and TFC_surface_ was the amount of flavonoids presented in the surface of FEMs.

### 2.6. Experimental Design

Response surface method (RSM) was employed to investigate the effect of independent parameters including MD to GA ratio, (MD : GA, *X*_1_), total solid content (*X*_2_), GSM content (*X*_3_), and core to coating ratio (core : coating, *X*_4_) on the process of microencapsulation. A Box-Behnken design (BBD) with four variables and three levels consisting of 29 experimental runs was constructed by the principal of RSM using the software of Design Expert V8.0.6. The range and levels of the independent parameters based on the preliminary experiment were presented in Table 1. The experimental points contain 24 factorial points and 5 center points (Table 2).

The experimental values were fitted under a second-order model in the form of quadratic polynomial equation [24]

(2) $$\begin{array}{c} Y=\beta_{0}+\overset{k}{\underset{i=1}{\sum }}\beta_{i}X_{i}+\overset{k}{\underset{i=1}{\sum }}\beta_{ii}X_{i}^{2}+\overset{}{\underset{i=1}{\sum }}\;\overset{}{\underset{j=1+1}{\sum }}\beta_{ji}X_{i}X_{j}+\varepsilon , \end{array}$$

where *Y* was response (EE, %). *β*_0_, *β*_*i*_, *β*_*ii*_, and *β*_*ji*_ were constant coefficients of intercept, linear, quadratic, and interaction terms, respectively. *X*_*i*_ and *X*_*j*_ were independent parameters.

### 2.7. Physicochemical and Morphological Analysis of FEMs

#### 2.7.1. Moisture Content

2 g of the sample was dried in an oven (SFG-02B, Huangshi, China) at 105°C until a constant weight, and the moisture content was calculated in terms of weight loss.

#### 2.7.2. Solubility

2 g of the sample was added into 50 mL of distilled water, and the mixture was agitated in a 100 mL glass beaker with a magnetic stirrer (C-MAG MS4, IKA, Germany) at 1000 rpm for 20 min [25]. Then the solution was centrifuged at 3000 rpm for 10 min. The residue was dried at 60°C and weighed. The solubility was measured by the decrease of weight.

#### 2.7.3. Bulk Density

The volume of weighed sample was determined using a graduated cylinder, and the bulk density was calculated by the quotient of weight and volume [26].

#### 2.7.4. Scanning Electron Microscope

Prior to scanning electron microscopy (SEM) analysis, FEMs were placed on a stub using double-side adhesive tape and then coated with gold. The analysis was carried out by using a scanning electron microscope Philips XL-30 ESEM (Netherlands) of low vacuum operated at 10 kV. Micrographs were taken at 1600x and 3200x, respectively.

### 2.8. *In Vitro* Antioxidant Activity of FEMs

#### 2.8.1. DPPH Radical Scavenging Assay

A 2.0 mL of sample at various concentrations (dissolved in 50% ethanol aqueous solution) was mixed with 2.0 mL of 200 *μ*M DPPH solution. The mixture was kept at room temperature for 30 min before measuring its absorbance at 517 nm [27]. Equation (3) shows the radical scavenging activity (RSA) formula:

(3) $$\begin{array}{c} \text{RSA}\left(\%\right)=\left[\frac{\left(A_{0}-A_{1}\right)}{A_{0}}\right]\times 100, \end{array}$$

where *A*_0_ was the absorbance of pure DPPH and *A*_1_ was the absorbance of DPPH in the presence of various extracts.

#### 2.8.2. Inhibition of Lipid Peroxidation

Lipid peroxidant value (POV) was measured according to Milovanovic et al. [28]. The sample was mixed with fresh lard. The lipid system was thoroughly homogenized (70 ± 0.5°C) for 30 min and stored at 65 ± 0.5°C in a water bath with stirring every 24 hr. POV was determined using Na_2_S_2_O_3_ titrimetric method:

(4) $$\begin{array}{c} \text{POV}\left(\text{meq}/\text{kg}\right)=\frac{S\times N\times 1000}{W}, \end{array}$$

where *S* was the volume of Na_2_S_2_O_3_, *N* was the normality of Na_2_S_2_O_3_, and *W* was the weight of sample. Fresh lard without antioxidant was used as a control.

### 2.9. Statistical Analysis

All analyses were performed in triplicate. Results were expressed as mean ± SD. Statistical analyses of the data were performed with one-way analysis of variance (ANOVA) or Student's *t*-test (SPSS 16.0). Significant differences (*P* < 0.05) between the means were determined using Tukey's multiple range test.

## 3. Results and Discussion

### 3.1. Optimization of Microencapsulation

#### 3.1.1. Response Surface Design (RSM) Model

As shown in Table 1, MD : GA (w/w), solid content (SC, %), GMS content (%), and core : coating (w/w) were investigated in the ranges of 1 : 1.5–1.5 : 1 (w/w), 20–30%, 0.2–0.4%, and 1 : 9–3 : 7, respectively. The response values (EE, %) ranged from 86 to 92% (Table 2) which were comparable to those reported in other literatures using gum Arabic or/and maltodextrins (Table 5).

After regression fitting, the quadratic equation expressing the relationship between EE (*Y*) and influence factors (*X*_*i*_) is modeled as follows:

(5) Y=90.61+0.90×X1+0.30×X2+0.55×X3 −2.16×X4+0.038×X1×X2−0.11×X1×X3 −0.52X1×X4+0.11×X2×X3+0.047×X2×X4 +0.16×X3×X4−1.15×X12 −0.38×X22−0.54×X32−1.18×X42.

**Figure 1:**
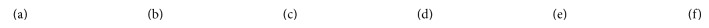
3D response surface plots for EE with respect to (a) MD : GA versus solid content; (b) MD : GA versus GMS content; (c) MD : GA versus core : coating; (d) solid content versus GMS content; (e) solid content versus core : coating; (f) GMS content versus core : coating.

In Table 3, the results demonstrated that the regression model could predict 97.43% of EE measured values (*P* < 0.0001, *R* ^2^ = 0.9743). The adeq precision of 21.046 (higher than 4) indicated that the model with an adequate noise ratio could be applied to this experimental design. *X*_2_, *X*_2_^2^, *X*_1_ *X*_4_ had significant effects on EE (*P* < 0.05) and *X*_1_, *X*_3_, *X*_4_, *X*_1_^2^, *X*_3_^2^, *X*_4_^2^ were highly significant (*P* < 0.01) and thus other forms of variables had negligible effects. Based on the regression coefficients and the *P* value, MD : GA (*X*_1_) and core : coating (*X*_4_) were the most critical factors to yield high EE, followed by total solid content (*X*_2_) and GSM content (*X*_3_). In addition, *X*_1_ and *X*_4_ were both extremely significant at first level (*P* < 0.0001) and second level (*P* < 0.001), indicating that minor changes of MD : GA and/or core : coating could affect the EE significantly. Adversely, total solid content (*X*_2_) and GMS content (*X*_3_) impacted EE more significantly at first level (*P* = 0.0214 for *X*_2_ and *P* = 0.003 for *X*_3_). The low *P* value of *X*_1_ *X*_4_ (*P* < 0.05) (Table 3) indicated the interactive effect of *X*_1_ and *X*_4_ (Figure 1(c)). At any given value of total solid (20–30%) or GMS content (0.2–0.4%), a decrease of MD : GA resulted in an increase of the EE (Figures 1(a), 1(b), and 1(c)) with the highest EE at MD : GA at 1 : 1.3 (w/w).

Overall, the condition with MD : GA at 1 : 1.3 (w/w), solid content at 27.4%, GMS content at 0.25%, and core : coating at 3 : 7 resulted in the maximum value of EE (91.75%).

#### 3.1.2. Maltodextrin to Gum Arabic Ratio (MD : GA)

In this study, MD : GA was identified to be critical factor to microencapsulate* R. tomentosa* flavonoids extract (*P* < 0.0001) with optimized ratio at 1 : 1.3 (w/w). Cilek et al. [29] described that MD to GA ratios from 10 : 0 to 3 : 2 increased the microencapsulation efficiency of phenolic compounds from sour cherry pomace after freeze drying process. Similarly, Idham et al. [19] also used 3 : 2 as MD : GA ratio to microencapsulate purified anthocyanin from* Hibiscus* resulting in optimal efficiency at 99.87% and high retention and stability of anthocyanin-rich microcapsules. However, previous research also showed that a high concentration of GA in the emulsion solution reduced encapsulation efficiency. Vidal et al. [30] described the decrease of encapsulation of maqui leaf extracts with more than 15% gum Arabic in emulsions (water-oil base) owing to the less solubility of plant extracts in the higher viscosity of coating material solutions. In addition, Tonon et al. [21] also reported that 6% of GA and 6% of MD (20 DE) demonstrated similar efficiencies on microencapsulating açai pulp (Table 5) which might be attributed by the fact that pectin and other polysaccharides in the açai pulp also acted as coating materials.

#### 3.1.3. Core to Coating Ratio (Core : Coating)

In contrast to MD : GA ratio, an increase of core : coating increased the EE (Figures 1(c), 1(e), and 1(f)) within our experimental limits (1 : 9 to 3 : 7), demonstrating a controversial result to that reported by Cilek et al. [29] who revealed that better encapsulation efficiencies were obtained when core : coating was 1 : 20 instead of 1 : 10. In the current study, the purified flavonoids extract was composed of myricetin (C_15_H_10_O_8_), quercetin (C_27_H_30_O_17_), dihydromyricetin (C_15_H_12_O_8_), kaempferol (C_15_H_10_O_6_), quercetin 7, 4′-diglucoside (C_27_H_30_O_17_), and vitexin (C_21_H_20_O_10_) according to our previous study [3]. The large amount of hydroxyl groups from flavonoids could rapidly form hydrogen bonds when presented in solution resulting in the formation of nonstarch polysaccharide-flavonoid complex* via* hydrogen bonding [31]. Among all the nonstarch polysaccharides, gum Arabic has been used mostly to form flavonoids-polysaccharide complex in the wine industry due to its high proportion of anion fraction contributed by glucuronic arabinogalactan [31]. In the light, gum Arabic could actively link to* R. tomentosa* flavonoids extract when they came in contact in the aqueous emulsion and was able to retain flavonoids extract throughout the spray drying process. The maltodextrin used in this study had 5–10 dextrose equivalents, which showed better retentions of flavors and polyphenols and higher yields while having a very low viscosity at high concentration [32, 33]. Therefore, the highly active interaction between* R. tomentosa* flavonoids extract and coating solution especially gum Arabic along with the functionality of maltodextrin (5–10 DE) contributed to the superior microencapsulating properties of the coating solution, which might explain that the higher the core : coating ratio the better the yield obtained in this study.

#### 3.1.4. Solid Content

The effect of solid content demonstrated weakest impacts among all the variables on the final encapsulation efficiency in this study (*P* = 0.0214) (Table 3). 27.4% of solid content in the emulsion was the optimal solid content to yield maximum encapsulation efficiency, agreeing with the results published by Robert et al. [17] in which in which 20.1% and 24.2% of maltodextrin yielded the optimal encapsulation of pomegranate juice (15.97°Brix) and pomegranate extract (13.80°Brix). In this study, the solid content higher than 27.4% generating the microcapsules with less encapsulated flavonoids extract might be explained by the reduction of carrier solubility resulting in reduction of encapsulated extract [34].

#### 3.1.5. Glycerol Monostearate (GMS)

GMS is a type of hydrophobic surfactant and foam stabilizer which were added to mango pulp (15 kg/1000 kg mango solid) and edible film formula (0.6%) before the drying processes [35, 36]. An appropriate percentage of GMS increased the interaction between flavonoids extract and coating solution as well as solubility and dispersibility of final microcapsules. However, because of the hydrophobic and foam inducing properties of GMS, higher concentration of GMS in the coating solution might adversely reduce the hydrogen bonding between flavonoids extract and coating material thus exposing more noncapsulated flavonoids extract on the surface of final powder which was indicated by the lower encapsulation efficiencies with the GMS percentage higher than 0.25%.

### 3.2. Physicochemical and Morphological Properties of FEMs

#### 3.2.1. Physicochemical Properties of Flavonoids Extract Microcapsules (FEMs)

As shown in Table 4, the color of FEMs prepared by the optimized conditions was creamy white. Bulk density was 0.35 g/cm^3^ comparable to the bulk densities reported in previous studies using maltodextrin and/or gum Arabic as coating materials and spray drying process [15, 20] (Table 5). Moisture content was 3.27% which was within the range of powder ingredients used in the food industry (3-4%) [37]. Consenting with the study by Pang et al. [38], high solid content (27.4%) in the feeding solution contributed to the low moisture content in the final FEMs. The high solubility (92.35%) of FEMs was contributed by hydrophilic properties of coating materials, mainly gum Arabic and maltodextrin, and also the exposure of hydrophilic groups on the FEMs surface after spraying drying [7]. In this study, FEMs possessed both high solubility and relatively high bulk density making it an ideal powder for food product application.

#### 3.2.2. Morphological Properties of FEMs

**Figure 2:**
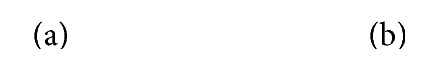
Scanning electron micrographs of FEMs prepared at optimal condition (a) 1600x magnification; (b) 3200x magnification.

Figure 2 presented the scanning electron microscopic photographs of FEMs. Most of the microcapsules were observed as irregularly spherical with some porous and dented spots on the surface. The formation of the dented surfaces was attributed to the shrinkage of the particles induced by the high temperature during spray drying process [39]. The morphology of FEMs was similar to other microcapsules manufactured through spray drying using gum Arabic and maltodextrin as coating materials [18, 29]. The irregularly sphere particle observed under SEM reflected on the high solubility and good bulk density of FEMs.

### 3.3. *In Vitro* Antioxidant Activity and Lipid Inhibition of FEMs

#### 3.3.1. DPPH Radical Scavenging Ability (DPPH-RSA)

**Figure 3:**
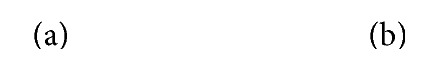
DPPH scavenging radical activity of (a) different concentrations of FEMs (b) 20 *μ*g/mL of FEMs, FE, rutin, V_C_, and citric acid. Data was shown as mean ± SD (*n* = 3).

After microencapsulation and spray drying, antioxidants were retained 95% in FEMs. As shown in Figure 3(a), the DPPH-RSA of FEMs increased with the increase of flavonoid concentrations, and the scavenging activity reached a maximum value (70.96%) at 20 *μ*g/mL. The ^•^DPPH-RSA of FEMs was also compared to other antioxidants including rutin, nonencapsulated flavonoids extract (FE), citric acid, and vitamin C (V_C_) at the same concentration of 20 *μ*g/mL (Figure 3(b)). RSA of FEMs was close to FE (73.27%), higher than rutin (35.15%) and citric acid (12.21%), and weaker than V_C_ (87.08%). Results indicated that, after microencapsulation process and high temperature spray drying, FEMs demonstrated similar DPPH-RSA as FE. Unlike the reduction of antioxidant activity observed in black carrot anthocyanins microcapsules [12], the process exerted little effect on the DPPH-RSA of FE from* R. tomentosa* which might be explained by the better thermal stability of flavonoids than that of anthocyanin.

#### 3.3.2. Inhibiting Ability of Lipid Peroxidation

**Figure 4:**
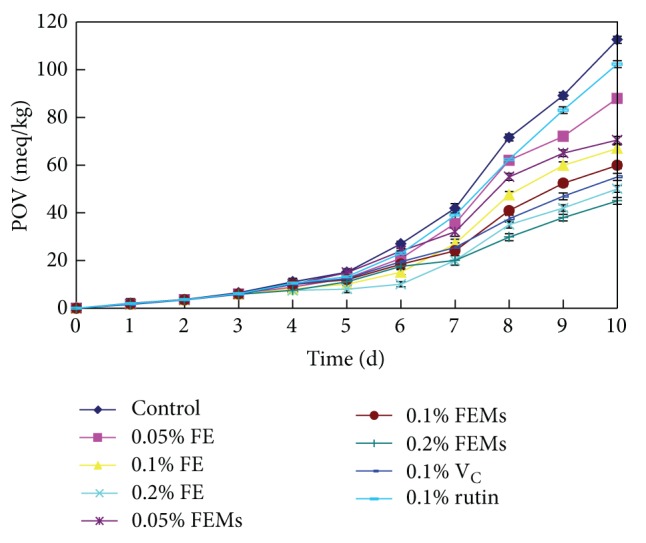
Inhibition effects of FEMs, FE, V_C_, and rutin on the lard peroxidation. Results were shown as means ± SD (*n* = 3).

The inhibiting ability of FEMs to lard peroxidation was investigated and compared with other antioxidants (Figure 4). As shown in control (Figure 4), under high temperature induction, the lipid peroxidation of lard was generated, and the POV value increased over time. The addition of antioxidants retarded the lipid peroxidation, thus slowing down the increment of POV value. Increase of FEMs significantly prevented lipid oxidation after 10 days of incubation (*P* < 0.05). Initially, POV values were higher under the FEMs treatments than those under the same concentrations of other antioxidants. However, after six days, lower POV values were tested in FEMs. At 0.1% level, V_C_ demonstrated the highest inhibiting capability followed by FEMs. Compared with FE, the coating of FEMs helped to prevent the oxidation of flavonoids caused by environmental factors (i.e., temperature, pH, and light) and slowly release lipid antioxidants to the system so that FEMs surpassed FE in inhibiting peroxidation. The slow release of antioxidant activities could associate with the high stability and long half-life time of bioactive compounds after being coated by gum Arabic or/and maltodextrin [18, 19, 21] (Table 5).

## 4. Conclusion

In this study, the microencapsulation conditions of flavonoids from the berries of* R. tomentosa* were optimized. Among maltodextrin to gum Arabic ratio, solid content, glycerol monostearate, and core to coating ratio, maltodextrin to gum Arabic ratio and core to coating ratio were identified as two critical factors and had interaction (*P* < 0.05). With 91.75% of encapsulation efficiency under the optimal condition, the FEMs were of antioxidant activities with good powder qualities in terms of bulk density, moisture content, and solubility. This study successfully accomplished the production of flavonoid rich microcapsules from* R. tomentosa* berries by spray drying at bench top scale. Future studies, however, need to investigate the stabilities of FEMs during storage and different food applications and also* in vivo* bioactivities as well.

## Figures and Tables

**Table 1:** Coded levels for independent variables used in experimental design for microencapsulation of FE.

Variables	Coded *X*_*i*_	Coded level		
		−1	0	1
MD : GA (w/w)	*X*_1_	1.5 : 1	1 : 1	1 : 1.5
SC (%)	*X*_2_	20	25	30
GMS content (%)	*X*_3_	0.2	0.3	0.4
Core : coating (w/w)	*X*_4_	3 : 7	2 : 8	1 : 9

FE, flavonoids extract; MD : GA, maltodextrin to gum Arabic ratio; SC, solid content; GMS, glycerol monostearate; core : coating, flavonoids extract to coating material ratio.

**Table 2:** Box-Behnken design for optimizing microencapsulation of FE.

Run number	Coded variable				Measured EE (%)
	*X*_1_	*X*_2_	*X*_3_	*X*_4_	
1	−1	−1	0	0	88.02
2	+1	−1	0	0	90.08
3	−1	+1	0	0	88.64
4	+1	+1	0	0	90.85
5	0	0	−1	−1	91.23
6	0	0	+1	−1	91.75
7	0	0	−1	+1	86.35
8	0	0	+1	+1	87.51
9	−1	0	0	−1	88.84
10	+1	0	0	−1	91.62
11	−1	0	0	+1	85.6
12	+1	0	0	+1	86.31
13	0	−1	−1	0	88.56
14	0	+1	−1	0	89.06
15	0	−1	+1	0	89.72
16	0	+1	+1	0	90.66
17	−1	0	−1	0	87.38
18	+1	0	−1	0	89.11
19	−1	0	+1	0	88.69
20	1	0	1	0	89.99
21	0	−1	0	−1	90.82
22	0	1	0	−1	91.12
23	0	−1	0	1	86.63
24	0	1	0	1	87.12
25	0	0	0	0	90.85
26	0	0	0	0	90.43
27	0	0	0	0	90.67
28	0	0	0	0	90.08
29	0	0	0	0	91.01

**Table 3:** Regression coefficients for the microencapsulation of FE values of regression coefficients calculated for the FE microencapsulation.

Source	Sum of squares	df	Mean square	*F* value	*P* value Prob > *F*	Significance
Model	86.42	14	86.42	324.69	<0.0001	∗∗
*X*_1_	9.70	1	9.70	87.72	<0.0001	∗∗
*X*_2_	1.09	1	1.09	570.17	0.0214	∗
*X*_3_	3.66	1	3.66	150.02	0.0003	∗∗
*X*_4_	55.73	1	55.73	2.16	<0.0001	∗∗
*X*_1_ *X*_2_	5.625*E* − 003	1	5.625*E* − 003	3.20	0.8552	
*X*_1_ *X*_3_	0.046	1	0.046	82.91	0.6026	
*X*_1_ *X*_4_	1.07	1	1.07	9.149*E* − 005	0.0225	∗
*X*_2_ *X*_3_	0.048	1	0.048	32.28	0.5942	
*X*_2_ *X*_4_	9.025*E* − 003	1	9.025*E* − 003	0.86	0.8173	
*X*_3_ *X*_4_	0.10	1	0.10	0.030	0.4410	
*X*_1_^2^	8.56	1	8.56	635.26	<0.0001	∗∗
*X*_2_^2^	0.94	1	0.94	378.51	0.0310	∗
*X*_3_^2^	1.88	1	1.88	2043.41	0.0043	∗∗
*X*_4_^2^	8.99	1	8.99	2095.49	<0.0001	∗∗
Residual	2.28	14	0.16			
*Lack of fit *	1.75	10	0.17	1.31	0.4287	
*Pure error *	0.53	4	0.13			
Cor total	88.70	28				

^*^ *P* < 0.05, significant;  ^**^ *P* < 0.01, highly significant.

**Table 4:** Color, bulk density, moisture content, and solubility of FEMs.

Color	Bulk density (g/cm^3^)	Moisture content (%)	Solubility (%)
Milk white	0.346 ± 0.013	3.27 ± 0.51%	92.35 ± 0.89%

Data were expressed as mean ± SD (*n* = 3).

**Table 5:** Representative literatures regarding microencapsulated plant extract/juice using gum Arabic and/or maltodextrin in spray drying process.

Plant extract	Formula of emulsion^*^	Drying condition	Resulted encapsulation	Reference
Purified flavonoids extract from *R. tomentosa* berries	Maltodextrin (5–10 DE) to gum Arabic ratio 1 : 1.3; solid content 27.4%; glycerol monostearate content 0.25%; core : coating material ratio 3 : 7.	40 MPa homogenization for 5 min; 0.7 mm diameter nozzle; 150°C inlet temperature and 100°C outlet temperature; 4.0 MPa atomization pressure; feeding rate 30%.	91.75% encapsulated flavonoids; milky white powder; irregularly sphere shape; particle size <20 *µ*m; 0.35 g/cm^3^bulk density; 3.27% moisture content; 92.35% solubility; 95% retention of DPPH radical scavenging activity; effectively retarded lipid oxidation.	This study

Pomegranate juice (15.97°Brix)	20.1% maltodextrin (12–20 DE) in coating solution; core : coating ratio 1 : 1.	1400 ×g homogenization speed for 5 min; 153°C inlet temperature; 600 L/h air flow; 10 mL/min feeding rate; 20 psi atomization pressure.	53.5% encapsulated polyphenols; 86.6% encapsulated anthocyanins; irregularly sphere shape.	[17]

Pomegranate ethanol extract (13.80°Brix)	24.3% maltodextrin (12–20 DE) in coating solution; core : coating ratio 2 : 1.	1400 ×g homogenization speed for 5 min; 153°C inlet temperature; 600 L/h air flow; 10 mL/min feeding rate; 20 psi atomization pressure.	71.0% encapsulated polyphenols; 82.0% encapsulated anthocyanins; irregularly sphere shape.	[17]

Black mulberry juice (40 kDa, adjusted to 11°Brix)	Maltodextrin (6 DE) to gum Arabic ratio 3 : 1; total solid 8%; 1.5% (w/w) microcrystalline cellulose in the juice.	130 inlet temperature; 800 L/h air flow rate; 925 N/m^2^aspiration rate; 20°C feeding temperature; 150 mL/h feeding rate; 65.3 psi atomization pressure.	82% drying yield; 87% solubility; 4.4 *μ*m particle size; 76.4°C glass transition temperature (Tg).	[15]

Anthocyanin ethanol extract from Cabernet Sauvignon (pH 2.4)	Maltodextrin (19 DE) to gum Arabic ratio 1 : 1.5; extract to maltodextrin ratio 1 : 1.	100 rpm homogenization speed for 2.5 hour; 150°C inlet temperature; 50°C outlet temperature; 25°C feeding temperature; 25% pump power; maximum aspirator rate.	Long half-life in soft drink; high retention of anthocyanins after 40 days of storage test; first-order kinetic degradation in soft drink; small and more sphere particles.	[18]

Purified anthocyanins extract from *Hibiscus sabdariffa* L. (8°Brix)	Maltodextrin (11–15 DE) to gum Arabic ratio 3 : 2; final solid content 20%.	14,000 rpm homogenization speed for 1 hour; 150°C, 500 feeding volume; 9.5% flow rate.	99.87% encapsulation efficiency; 3.7% degradation rate at 4°C; strong color stability; high retention of anthocyanins over 3-month periods; 6.2 and 5.4 months half-life under 4°C and 25°C, respectively.	[19]

Filtered açai pulp (3% solid content)	6% maltodextrin 10 DE in the pulp.	140°C inlet temperature; 78°C outlet temperature; 73 mL/h air flow and 0.06 MPa compressor air pressure; 15 g/min feed flow rate; 35°C storage temperature.	0.39 g/mL bulk density; 1.531 g/mL absolute density; 74.5% porosity; 3436.85 mg/100 juice dried matter; 1165.84 *μ*mol TE/g juice dried matter; 80% retention of antioxidant activity under 0.328 water activity and 35°C storage condition.	[20]

Filtered açai pulp (3% solid content)	Two best formulas: (1) 6% maltodextrin 20 DE in the pulp and (2) 6% gum Arabic in the pulp.	140°C inlet temperature; 78°C outlet temperature; 73 mL/h air flow and 0.06 MPa compressor air pressure; 15 g/min feed flow rate.	(i) 20 DE maltodextrin formula: 2.88% moisture content, 0.245 water activity; 96.12% solubility; 19.69% hygroscopicity; 9.33 *μ*m particle size; 100% retention of total polyphenolics and antioxidant activity after 15 days in both high and low water activity environments. (ii) Gum Arabic formula: 3.04% moisture content; 0.244 water activity; 94.78% solubility; 19.74% hygroscopicity; 9.41 *μ*m particle size; 100% retention of total polyphenolics and antioxidant activity after 15 days in both high and low water activity environments.	[21]

Ethanolic anthocyanin rich extracts from black carrot (6% solid content)	Glucodry 210 (20–23 DE); 20% of final solid content in the emulsion.	160°C inlet temperature; 107°C outlet temperature; 120% pump power; 5 mL/min feeding rate; 25°C feeding mixture.	630 mg/100 g anthocyanin content; 17.12 mg sample/mg DPPH EC_50_ antiradical activity; 6.03% moisture content; 76.64% hygroscopic moisture; high retention of anthocyanins (84% at 4°C and 67% at 25°C) after storage 64 days.	[12]

^*^Formula of emulsion stands for the optimal formula for microencapsulation if optimization study was conducted.
